# Cyclosporine A-Loaded Ternary Solid Dispersion Prepared with Fine Droplet Drying Process for Improvement of Storage Stability and Oral Bioavailability

**DOI:** 10.3390/pharmaceutics15020571

**Published:** 2023-02-08

**Authors:** Tatsuru Moritani, Hayato Usui, Tadahiko Morinaga, Hideyuki Sato, Satomi Onoue

**Affiliations:** 1 Laboratory of Biopharmacy, School of Pharmaceutical Sciences, University of Shizuoka, 52-1 Yada, Suruga-ku, Shizuoka 422-8526, Japan; 2 Material and Advanced Technology Development Center, Innovation/R&D Division, RICOH Company, Ltd., 2-7-1 Izumi, Ebina 243-0460, Japan

**Keywords:** cyclosporine A, fine droplet drying process, mucoadhesive particle, oral absorption, storage stability, ternary solid dispersion

## Abstract

This study aimed to develop a cyclosporine A (CsA)-loaded ternary solid dispersion (tSD/CsA) to improve the storage stability of a solid dispersion (SD) system and the oral absorbability of CsA. Hydroxypropyl cellulose (HPC) and hydroxypropyl methylcellulose acetate succinate (HPMCAS) were selected as carrier materials of tSD, and tSD/CsA was prepared with a fine droplet drying process, a powderization technology that employs an inkjet head. The physicochemical properties of tSD/CsA were evaluated in terms of morphology, storage stability, dissolution behavior, and mucoadhesive property. After the oral administration of CsA samples (10 mg-CsA/kg) to rats, the plasma concentration of CsA was monitored to estimate oral absorbability. tSD/CsA comprised uniform shriveled particles with a diameter of 3.4 mm and span factor of 0.4, which is a parameter to estimate the particle size distribution. Although HPC-based binary SD showed marked aggregation of the particles after storage under 40 °C/75% relative humidity, there were no significant aggregations of tSD/CsA, due to the relatively low hygroscopic property of HPMCAS. The pH-dependent release of CsA with improved dissolution was observed in tSD/CsA. In the in vitro mucoadhesive evaluation using a mucin disk, tSD/CsA exhibited a better mucoadhesive property than HPC-based SD, possibly leading to prolonged retention of tSD particles in the gastrointestinal tract after oral administration. Orally-dosed tSD/CsA in rats resulted in significantly improved oral absorption of CsA, as evidenced by a 27-fold higher bioavailability than amorphous CsA. tSD/CsA may be a promising dosage option to improve the storage stability of a SD system and the biopharmaceutical properties of CsA.

## 1. Introduction

Recently, several printing technologies have been applied to the production of pharmaceutical formulations in the form of particles, hydrogels, and films due to their high standards of being flexible, robust, and reliable [1,2]. The fine droplet drying (FDD) process, a novel powderization technique using inkjet technology, was developed for designing functional micron-sized particles by our group [3,4]. One of the characteristics of inkjet heads is their ability to generate uniform-sized droplets with high efficiency and strict controllability of the particle size compared with the conventional spray nozzle (e.g., two-fluid nozzle and rotary atomizer) to achieve high-quality printing. In a previous study, the FDD process was first applied for the development of a solid dispersion (SD) to improve the oral bioavailability of cyclosporine A (CsA) [3]. The SD system, defined as a dispersion of one or more active ingredients in an inert carrier or matrix in a solid state, is a representative technique to improve the water solubility of poorly water-soluble compounds by enhancing wettability, dispersibility, and amorphization [5]. From previous work, although the hydroxypropyl cellulose (HPC)-based SD formulation prepared by the FDD process successfully improved the physicochemical properties and oral bioavailability (BA) of CsA in rats, there were stability problems during storage under accelerated conditions, including the aggregation of particles and solubility reduction. This kind of problem related to the storage stability of formulations makes it challenging to develop commercial SD formulations, suggesting the importance of particle design and polymer selection to achieve desirable physicochemical stability [6,7].

For the production of SD systems, hydrophilic polymers like polyethylene glycol, hydroxypropyl methyl cellulose, Soluplus^®^, polyvinylpyrrolidone, and HPC are typically selected as carrier materials to improve dispersibility and wettability, leading to the enhancement of the dissolution characteristics and subsequently oral BA. Such polymers often have a hygroscopic character, possibly causing significant changes in physicochemical properties such as particle size, solubility, dissolution properties, and crystallinity during the storage period [3,8,9]. For further improvement of their performance in terms of storage stability and processability, a recent advancement of the SD approach is to design ternary solid dispersions (tSD) [10]. Along with the drug and single carrier material for the conventional binary system, there are other components of the tSD system like a second polymer, surfactant, and excipients, and the addition of a third component can functionalize the SD system depending on the physicochemical characteristics of that component, including the prevention of precipitation, improvement of storage stability, and controlled release of included drugs in addition to solubility enhancement [11,12,13,14]. These findings prompted us to apply the tSD strategy to the previous HPC-based SD system, and hydroxypropyl methylcellulose acetate succinate (HPMCAS) as the third component to improve and control physicochemical properties and oral BA of CsA due to its enteric, low hygroscopic, and mucoadhesive properties, possibly contributing to the functionalization of the HPC-based binary SD system.

The aim of this study was to clarify the applicability of the FDD process to design a tSD system and evaluate the formulation, consisting of HPMCAS and HPC, and CsA (tSD/CsA). Cellulose derivatives have been known to not only inhibit drug crystallization in the SD system but also act as mucoadhesive polymers [15], expecting enhancement of oral BA due to prolonged retention in the gastrointestinal tract. In this context, tSD/CsA could achieve the enhancement of the dissolution behavior of CsA with pH-dependent release, and improved storage stability and oral BA. The stability study was conducted to estimate the physicochemical differences between tSD/CsA and a conventional binary SD system. The pharmacokinetic characteristics were evaluated to assess the possible enhancement of absorbability after oral administration.

## 2. Materials and Methods

### 2.1. Materials

CsA and HPC-SSL were purchased from Tokyo Chemical Industry Co., Ltd. (Tokyo, Japan) and FUJIFILM Wako Pure Chemical Co. (Osaka, Japan), respectively. HPMCAS-HG was kindly supplied by Shin-etsu Chemical Co., Ltd. (Tokyo, Japan). All other reagents were purchased from commercial sources.

### 2.2. Preparation of tSD/CsA

tSD/CsA was prepared by a FDD process in accordance with the reported preparation method with some modifications [3]. Briefly, amorphous CsA (100 mg) and carrier polymers [HPMCAS-HG (950 mg) and HPC-SSL (950 mg)] were dissolved in acetone, with a total solid content of 2% (*w*/*w*). The solution was atomized by MH2420 (RICOH, Tokyo, Japan) (driving frequency and voltage of piezo element: 310 kHz and 10 V), and powderized by dried air under at 24 °C with a flow rate of 50 m^3^/h. HPC-SSL-based SD of CsA (SD/CsA) (5% CsA loading) was prepared by the same method as described above using 1,4-dioxane as an organic solvent.

To estimate the phase separation of each polymer during the drying process, a thin layer film was prepared by the film casting method on a glass slide. The prepared film was dried at 30 °C for 3 h, and observed by optical microscope.

### 2.3. Physicochemical Characterization

The surface morphology of CsA samples was evaluated by scanning electron microscopy (SEM) using Miniscope^®^ TM3030 (HITACHI, Tokyo, Japan) with a 10 nm platinum coating by a magnetron sputtering device (MSP-1S, Vacuum device, Ibaraki, Japan). Laser diffraction analysis was carried out using Microtrac MT3000II (MicrotracBel, Osaka, Japan) with compressed air (0.2 MPa). The span factor was calculated with the following equation: SPAN = (d_90_ − d_10_)/d_50_, where d_10_, d_50_, and d_90_ are the diameters of particles at 10, 50, and 90%, calculated as a volume, respectively. The X-ray powder diffraction (XRPD) patterns of CsA samples were collected with MiniFlex II (Rigaku, Tokyo, Japan) with Cu Kα radiation generated at 15 mA and 40 kV. Data were obtained from 4° to 40° (2*θ*) at a step size of 0.2° and scanning speed of 2°/min.

The dissolution behaviors of CsA samples in first fluid (pH 1.2) and second fluid (pH 6.8) for the dissolution test, which were prepared in accordance with Japanese Pharmacopeia 17th edition, were evaluated using NTR 6100A (Toyama, Sangyo, Osaka, Japan) under stirring at 50 rpm and 37 °C. Amorphous CsA, tSD/CsA, and SD/CsA (ca. 2 mg-CsA) were weighed and added into the glass beaker with 100 mL of dissolution media. Collected samples were filtered through a 0.2-μm mesh and diluted in equal amounts of acetonitrile as evaluation samples. The determination of the CsA concentration was conducted using a Waters Acquity UPLC system in accordance with Section 2.6.

### 2.4. Storage Stability Studies

To estimate the physicochemical differences between tSD/CsA and SD/CsA under accelerated conditions, the samples were kept in a stability chamber (SRH-15VEVJ2, Nagano Science Co., Ltd., Osaka, Japan) for a week at 40 °C/75% RH. After the storage, the aged samples were physicochemically characterized in terms of morphology, particle size distribution, and crystallinity.

### 2.5. Mucoadhesive Property

The mucin disk method was used to evaluate the mucoadhesive properties of prepared formulations according to the reported method with slight modifications [16]. Briefly, 400 μL of 10% mucin solution was spread on a 25 mm diameter filter paper, and the paper was dried at ambient temperature. Five hundred microliters of suspensions of each formulation (10 mg/mL) in phosphate buffer (pH 6.8) were spread on the mucin disk, and the disk was dried at room temperature for 5 min. Each mucin disk with sample was fixed to a glass slide, immersed in 40 mL of phosphate buffer at 37 °C, and incubated for 30 min. After the incubation, the amount of CsA remaining on the disk was determined using the UPLC system.

### 2.6. Determination of CsA Amount

To determine the amount of CsA in all experiments, including in vitro and in vivo experiments, a Waters Acquity UPLC system (Waters Corporation, Milford, MA, USA) equipped with a single quadrupole mass detector was used with the analytical conditions reported previously [3].

### 2.7. Pharmacokinetic Study in Rats

For pharmacokinetic study, Sprague–Dawley rats weighing 164.4 ± 5.2 g (Japan SLC INC., Shizuoka, Japan) were used. All animal experiments were approved by the Institutional Animal Care and Ethical Committee of the University of Shizuoka (Approval No. 196367). CsA samples (10 mg-CsA/kg) were suspended in distilled water (2 mL) and administered to rats. To calculate absolute oral bioavailability, pharmacokinetic study was conducted after intravenous administration of CsA (0.5 mg/kg) dissolved in DMSO. After the administrations, the blood samples (200 µL) were collected at each time point and centrifuged at 10,000× *g* for 10 min at 4 °C to obtain plasma. Acetonitrile with 5 μg/mL of tamoxifen (as internal standard) (150 µL) was used for the deproteinization of the plasma samples (50 μL). Precipitated protein was then separated by centrifugation at 10,000× *g* for 10 min at 4 °C, and filtered through a 0.2-μm filter. The prepared samples were analyzed by an internal standard method using the UPLC system described in Section 2.6.

### 2.8. Statistical Analysis

The experimental data were statistically analyzed using one-way analysis of variance (ANOVA) followed by Tukey’s multiple comparison. A *p*-value less than 0.05 was considered to be statistically significant for all experiments.

## 3. Results and Discussion

### 3.1. Appearance and Particle Size Distribution of tSD/CsA

**Figure 1:**
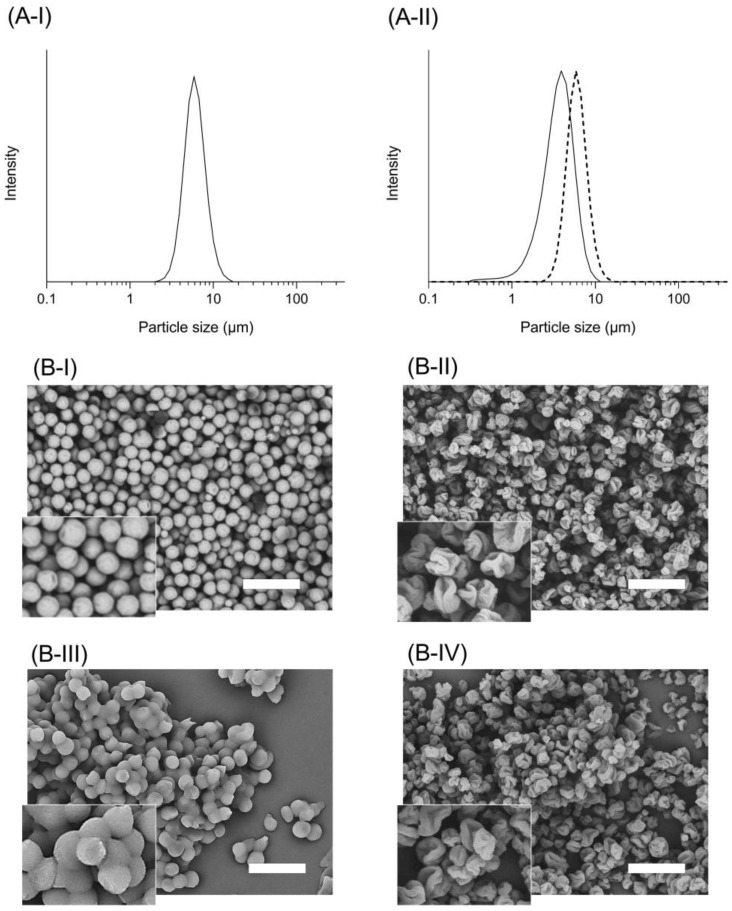
Particle size distribution and appearance of CsA-loaded solid dispersions. (**A**) Laser diffraction analysis of CsA samples to measure the particle size distribution. (**A-I**) SD/CsA and (**A-II**) tSD/CsA. Solid line, particle size distribution before storage; and dotted line, particle size distribution after storage at 40/75%RH for one week. (**B**) SEM observations of CsA samples. (**B-I**) SD/CsA and (**B-II**) tSD/CsA. (**B-III**,**B-IV**) after storage at 40/75%RH for one week. White bars represent 15 μm.

In the present study, the FDD process was used to prepare tSD/CsA and SD/CsA, and the overall yields of tSD/CsA and SD/CsA were over 75%, respectively. The particle sizes of tSD/CsA and SD/CsA measured by laser diffraction analysis were 3.4 and 5.6 μm (Figure 1A), and SPAN factors were calculated as 0.4 and 0.7, respectively. As evidenced by the calculated SPAN factors, both formulations had uniform particle size distributions, since the inkjet head used in the FDD process could provide highly uniform micron-sized droplets [3]. The obtained particles were visualized by SEM observation to clarify their morphologies (Figure 1B). In the SEM images, SD/CsA was almost spherical with a uniform particle size distribution, whereas tSD/CsA appeared to be shriveled particles. The difference in surface morphology between tSD/CsA and SD/CsA would be partly explained by the influence of the evaporation rate of the organic solvent. In this study, acetone and 1,4-dioxane were utilized as solvents to prepare tSD/CsA and SD/CsA, respectively. On the basis of classification of residual solvents in accordance with ICHQ3C guidelines, 1,4-dioxane and acetone are classified as a class 2 and class 3 solvent, respectively. With respect to the quality control of manufacturing, these solvents should be limited and controlled under the concentration limit due to their toxicity. The boiling points and vapor pressures of acetone and 1,4-dioxane are 56 and 101 °C at 1 atm, and 35 and 231 mmHg at 298 K, respectively. A high evaporation rate could lead to the rapid generation of a shell structure on the droplet surface, possibly creating a void within the particles and the subsequently shriveled surface after removing the inner solvent. Such differences in particle shape could also be caused by phase separation and polymer localization on the surface of ternary systems [17,18]. In the case of tSD/CsA, one of the polymers is considered to be enriched on the particle surface, potentially leading to the wrinkled particles depending on the polymer properties, such as their solubility, mobility, and miscibility. Although the difference in the evaporation rate of organic solvent might influence the crystallinity of generated particles, both tSD/CsA and SD/CsA were in an amorphous state after the drying process (Figure S1), possibly contributing to the improved dissolution behavior of CsA.

Previously, evaporation-induced self-assembly led to phase separation among different components during the spray-drying process, resulting in the core-shell spatial configuration depending on the diffusiveness of the used polymers [19]. According to the estimation of phase separation by film casting of HPC-SSL, HPMCAS, and HPC-SSL: HPMCAS (1:1) solution, polymer films composed of a single polymer were uniform and translucent, whereas the mixed polymer film was obviously turbid (Figure S2A). The images obtained by optical microscope showed the uniform translucent structure of polymer films composed of HPC-SSL or HPMCAS-HG. On the other hand, the mixed polymer film exhibited a sea-island structure possibly due to the phase separation of HPC-SSL and HPMCAS during the drying process (Figure S2B). According to the physicochemical properties of HPC-SSL and HPMCAS, HPMCAS might compose the surface of tSD/CsA particles owing to the faster precipitation in the drying process caused by the hydrophobicity and poor aqueous solubility. Considering these points, phase separation of polymers on the surface of tSD/CsA could lead to a maldistribution of polymer composed of tSD/CsA, possibly due to the different diffusiveness of polymers in the solution, contributing to the controlled drug release and enhanced storage stability of tSD particles.

### 3.2. Storage Stability of tSD/CsA

Stability problems during the storage period have markedly hampered development of the SD formulations due to the high energy state and hygroscopic property of selected polymers [20]. To evaluate the storage stability of tSD/CsA and SD/CsA, these samples were stored under 40 °C/75%RH, and evaluated with respect to changes in the size distribution, surface morphology, and crystallinity. After the storage of SD/CsA, there were significant changes in appearances between fresh and aged micro particles in the SEM image (Figure 1(B-I,B-III)), and it was difficult to measure the size distribution by laser diffraction analysis even using compressed air owing to the severe aggregation. This particle adhesion was mainly caused by moisture absorption of HPC-SSL due to its high hygroscopic property [21]. Moisture absorption could influence not only the powder property (i.e., particle appearance and size distribution) but also solubility due to the transformation of the crystalline form [22]. However, the appearance and particle size distribution of tSD/CsA were not significantly changed even after storage (Figure 1(A-II,B-II,B-IV)). The existence of HPMCAS might help to prevent the aggregation of tSD particles, possibly due to the relatively low hydrophilic property of HPMCAS. When highly-wettable material like a hydrophilic polymer is exposed to humid air, the polymer can absorb water, which plasticizes the polymer, increasing its mobility, causing a decrease in its glass transition temperature (Tg), and subsequently leading to the alteration of physicochemical properties [23]. However, the hydrophobicity of HPMCAS results in much less absorption of water than that observed for typical water-soluble polymers. The relatively high Tg value of HPMCAS directly correlates with the drug, showing low mobility within the SD system, which is responsible for improved physical stability. HPMCAS on the tSD/CsA surface could inhibit the water sorption and softening of HPC-SSL within tSD/CsA, resulting in a higher storage stability compared with SD/CsA stored even under 40 °C/75%RH. Although tSD/CsA and SD/CsA are not final dosage forms and some additional excipients would be necessary to optimize the formulation composition, tSD might contribute to designing optimal formulation with high storage stability by adding minimal excipients due to its low hygroscopic property. Generally, there are stability concerns on crystallinity of included drug within SD formulation due to the high free energy of amorphous state [24]. According to the data from an XRPD analysis of tSD/CsA and SD/CsA (Figure S1), there were no significant changes in crystallinity even after the storage under 40 °C/75% RH.

### 3.3. Dissolution Behavior

**Figure 2:**
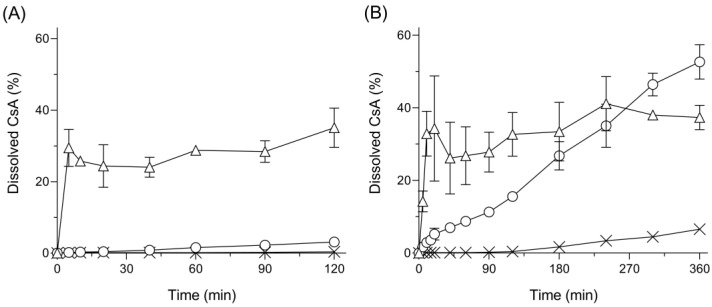
Dissolution behavior of CsA samples in (**A**) pH1.2 and (**B**) pH6.8 solutions. ×, amorphous CsA; ◯, tSD/CsA; △, SD/CsA. Each bar represents mean ± S.E. of three independent experiments.

The dissolution testing of amorphous CsA, tSD/CsA, and SD/CsA was performed under pH 1.2 and 6.8 conditions to mimic the pH in the GI tract (Figure 2). CsA can be classified as a biopharmaceutics classification system (BCS) class II compound [25]; therefore, amorphous CsA showed poor dissolution properties under both pH conditions due to its poor water solubility. Conversely, even at 5 min after starting the dissolution test, SD/CsA exhibited rapid dissolution of CsA at both pH 1.2 and 6.8, as evidenced by 100- and 260-fold higher dissolution, respectively, compared with that of amorphous CsA (Figure 2A,B). In the case of tSD/CsA, there were significant differences between dissolution kinetics at pH1.2 and 6.8. Under the pH1.2 condition, the dissolution rate was very slow and the dissolved amount fo CsA was only 0.6 mg/mL after 120 min. On the other hand, under pH 6.8, although tSD/CsA showed an improvement of the dissolution amount of CsA as well as SD/CsA, a gradual release of CsA from tSD/CsA was observed. HPMCAS, the third component of tSD/CsA and known as a pH-responsive polymer, was not dissolved under the acidic condition; thus, HPMCAS is commonly used as a carrier for enteric formulations [23]. Due to the presence of relatively hydrophobic methoxy and acetate substituents, HPMCAS is water-insoluble when un-ionized (about pH < 5) and remains predominantly colloidal at an intestinal pH (pH 6.0–7.5). Considering the clear differences in dissolution behavior, the surface of tSD/CsA could be mainly covered with HPMCAS caused by the phase separation described in Section 3.2. In addition to the enhancement of storage stability, the tSD system could control the release behavior of included drugs with sustained-release kinetics depending on the pH, possibly contributing to the control of oral absorption behavior from the GI tract.

### 3.4. Mucoadhesive Properties

**Figure 3:**
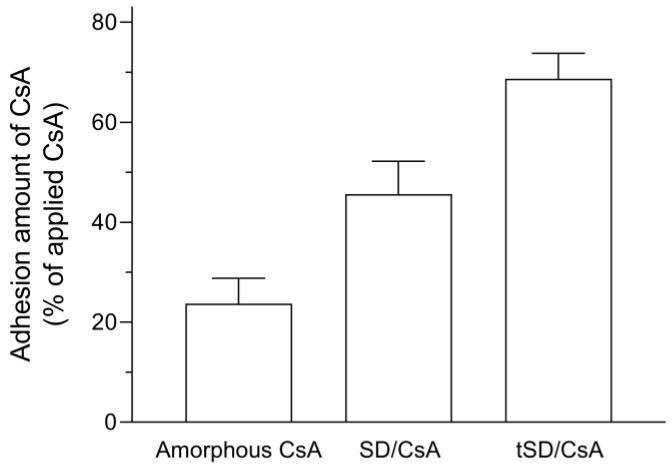
Mucoadhesive property of CsA samples. Amount of CsA retained after the application of CsA samples to mucin disks. Each bar represents mean ± S.E. of three independent experiments.

The mucoadhesive properties of tSD/CsA and ASD/CsA were evaluated using the mucin disk method reported previously [16]. CsA amounts of SD/CsA and tSD/CsA were higher than that of amorphous CsA, as evidenced by 2- and 3-fold residual amounts of CsA, respectively (Figure 3). According to previous reports, hydrogen-bonding groups (-OH, -COOH, etc.), anionic/anionic charges, and a high molecular weight can contribute to the mucoadhesive properties between a mucus layer and carrier polymers due to their electrostatic interactions and molecular entanglements [26]. Considering the chemical structures of carrier polymers, both HPC-SSL and HPMCAS have a hydroxy group and/or carboxyl group within their chemical structures, possibly leading to electrostatic interactions between mucin and polymers. In the case of tSD/CsA, there was a 1.5-fold amount of CsA compared with SD/CsA, suggesting better mucoadhesive properties of tSD particles. The difference in the residual amount of CsA on the mucin disk between tSD/CsA and SD/CsA could be partly explained by the physical states of each particle under the test conditions during the mucin disk method. As the results of the dissolution tests indicate, the concentration of CsA in SD/CsA was rapidly increased compared with that of CsA in tSD/CsA at both pH1.2 and 6.8, suggesting the quick dissolution of carrier polymer and dispersion of CsA molecules after SD/CsA particles were introduced into the water. In this context, tSD/CsA exhibited the gradual release of CsA under the pH6.8 condition owing to the existence of HPMCAS within the tSD system. This slow diffusion of CsA could increase the retention of tSD particles onto the mucin disk, leading to a relatively high amount of CsA in intestinal tissue. From these results, tSD/CsA has the potential to lead to higher BA than SD/CsA due to its mucoadhesive property.

### 3.5. Pharmacokinetic Profiles after Oral Administration in Rats

**Figure 4:**
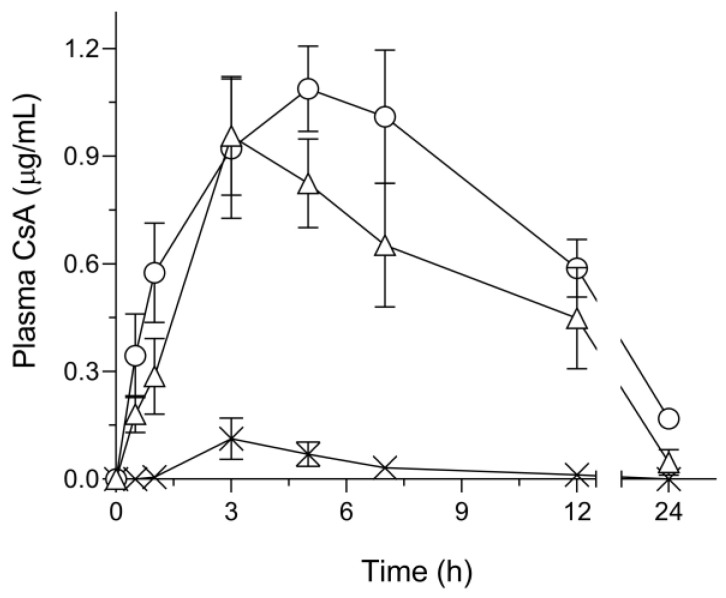
Oral absorption profile of CsA samples after oral administration (10 mg-CsA/kg, p.o.) in rats. ×, amorphous CsA; ◯, tSD/CsA; and △, SD/CsA. Each bar represents mean ± S.E. of 5–6 independent experiments.

To evaluate the possible improvement of the oral absorption behavior of CsA, a pharmacokinetic study was conducted after oral administration of CsA samples in rats (Figure 4). Pharmacokinetic parameters including the area under the curve of blood concentration versus time curve (AUC_0–inf_.), BA, and mean residence time (MRT) were calculated from the blood concentrations of CsA (Table 1). There was poor oral absorption of CsA in the amorphous CsA group, as evidenced by AUC_0–inf_. and BA of 0.55 μg·h/mL and 0.7%, respectively. This poor absorbability of CsA corresponded with the results of the dissolution tests of amorphous CsA. AUC_0–inf_. and BA of SD/CsA were 10 μg·h/mL and 13%, respectively, and those of tSD/CsA were 15 μg·h/mL and 19%, respectively. In terms of oral absorption properties, these SD formulations clearly improved the absorbability compared with amorphous CsA (i.e., BA of SD/CsA and tSD/CsA were 19-fold and 27-fold higher, respectively) due to the improved dissolution behavior of CsA. CsA can be classified as a BCS class II drug characterized by poor solubility and high membrane permeability, so that improved dissolution characteristics can significantly enhance its absorbability [3]. With respect to the residence time of CsA after oral administration of CsA samples, the MRT value in the tSD/CsA group was 1.2-fold higher than that of SD/CsA. In addition, the time to maximum concentration (T_max_) of tSD/CsA was also longer compared with that of SD/CsA. These results could be partly explained by the sustained release profile of CsA from tSD/CsA particles observed in the dissolution test and interaction between the mucus layer and tSD/CsA. Regarding the interaction on the mucosal surface, it has been reported that a mucoadhesive formulation consisting of cellulose derivatives was developed to achieve a prolonged duration of action, and it led to a higher BA of tolmetin sodium compared with commercial capsules [27]. In the present study, the mucoadhesive properties of tSD/CsA could contribute to the improved oral absorption of CsA. Additionally, a high concentration of CsA on the surface of the absorption site might be achieved by the mucoadhesion of tSD/CsA, and the simple diffusion process of CsA through the epithelial membrane of the intestine could be enhanced according to Fick’s law [28], possibly contributing to the higher C_max_ compared with that of SD/CsA. From these findings, HPMCAS-HG might play a beneficial role in improving the pharmacokinetic behavior of CsA in tSD/CsA derived from sustained release behavior and mucoadhesive properties.

## 4. Conclusions

The CsA-loaded tSD system prepared by the FDD process showed high storage stability and mucoadhesive properties. tSD/CsA exhibited pH-dependent gradual drug release with an improved dissolution behavior at pH1.2 and 6.8, possibly derived from the characteristics of HPMCAS. In the pharmacokinetic study, the oral absorption efficiency of tSD/CsA was enhanced compared with that of SD/CsA due to its mucoadhesive property and improved dissolution characteristics. On the basis of these findings, the tSD system may be a viable formulation strategy to improve storage stability and pharmaceutical properties.

## Figures and Tables

**Table 1:** Pharmacokinetic parameters of CsA after oral administration of CsA samples (10 mg/kg) to rats.

	C_max_ (μg/mL)	T_max_ (h)	MRT (h)	AUC_0–inf_. (μg·h/mL)	BA (%)
Amorphous CsA	0.12 ± 0.057	3.4 ± 0.40	5.9 ± 0.47	0.55 ± 0.22	0.73
tSD/CsA	1.3 ± 0.080	5.8 ± 0.49	8.7 ± 0.31	15 ± 1.3	19
SD/CsA	0.97 ± 0.17	3.8 ± 0.49	7.5 ± 0.84	10 ± 2.0	13

## Data Availability

Not applicable.

## Supplementary Materials

The following supporting information can be downloaded at https://www.mdpi.com/article/10.3390/pharmaceutics15020571/s1, Figure S1: crystallinity assessment of CsA samples; Figure S2: phase separation characteristics visualized by film casting method.
