# Supplementary material for: Cyclosporine A-Loaded Ternary Solid Dispersion Prepared with Fine Droplet Drying Process for Improvement of Storage Stability and Oral Bioavailability
Source: Pharmaceutics. 2023 Feb 8;15(2):571. doi: 10.3390/pharmaceutics15020571 (PMC9965122; doi:10.3390/pharmaceutics15020571)
Supplement: Supplementary file 1 [file pharmaceutics-15-00571-s001.zip › pharmaceutics-2145489-supplementary.pdf]

Supplementary information

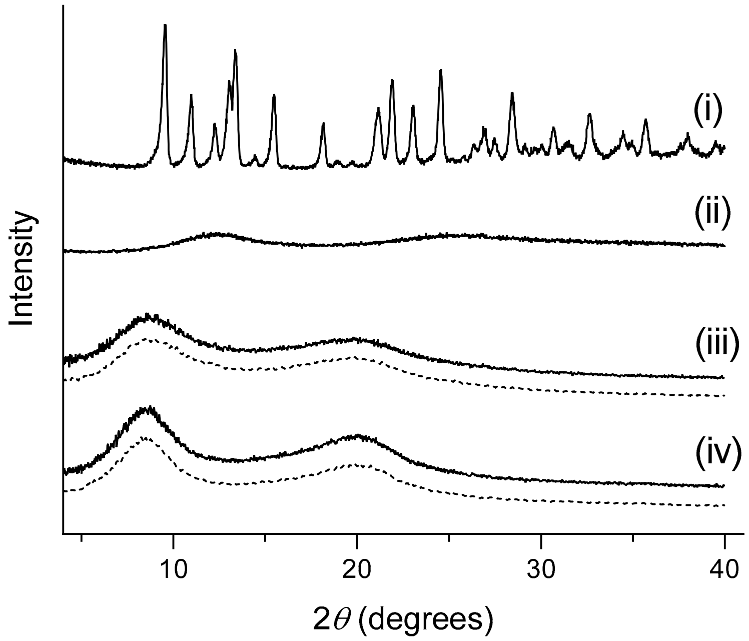

Figure S1. Crystallinity of CsA samples evaluated by X-ray powder diffraction analysis. (i) Crystalline CsA, (ii) amorphous CsA, (iii) tSD/CsA, and (iv) SD/CsA (solid line, initial samples; and dashed line, 1 week-aged samples under 40°C/75%RH).

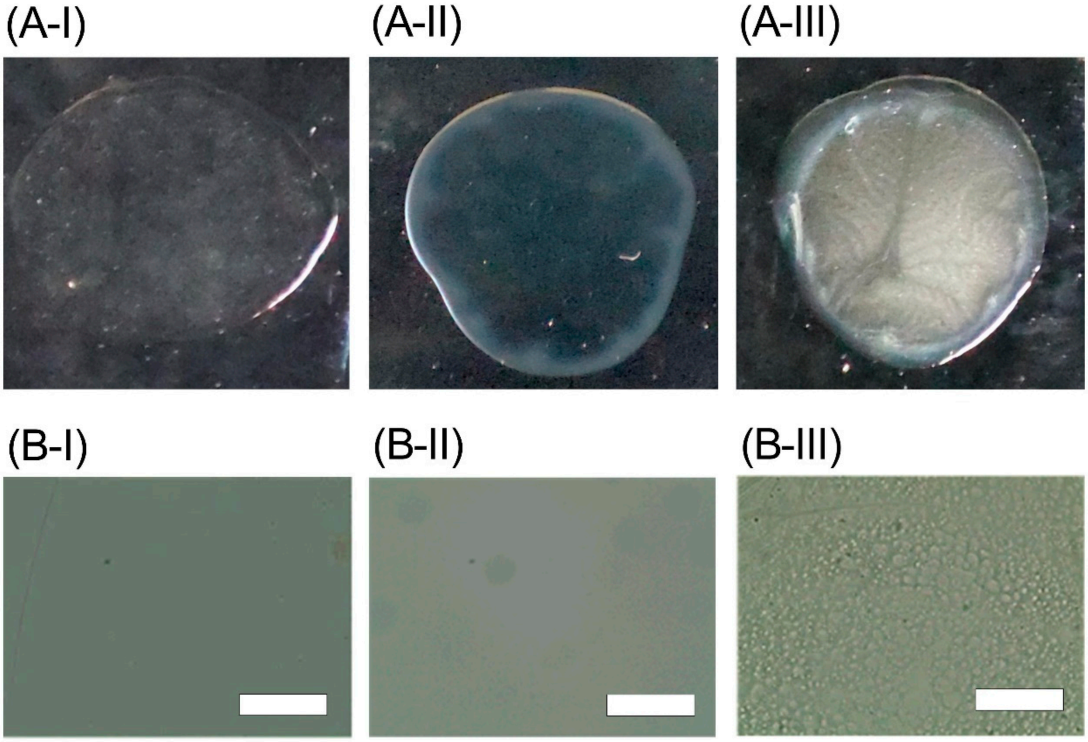

Figure S2. Appearance of polymer films prepared by film casting method on a slide grass evaluated by (A) visual observation and (B) optical microscope. (I) HPC-SSL, (II) HPMCAS-HG, and (III) mixture of HPMCAS-HG and HPC-SSL. White bars represent 100 μm.
